# Genome-Wide Resequencing Reveals High Connectivity and Localized Adaptive Signals in Manila Clam (*Ruditapes philippinarum*) Populations Along the Southeastern Coast of China

**DOI:** 10.3390/ani16121897

**Published:** 2026-06-18

**Authors:** Yatong Yao, Yaoran Fan, Shuaijie Wang, Yanming Sui, Baojun Tang, Zhiguo Dong, Hanfeng Zheng

**Affiliations:** 1 East China Sea Fisheries Research Institute, Chinese Academy of Fishery Sciences, Shanghai 200090, China; yaoyt@ecsf.ac.cn (Y.Y.); fanyrhh@163.com (Y.F.); wangsj@ecsf.ac.cn (S.W.); bjtang@yeah.net (B.T.); 2 College of Fisheries and Life Science, Dalian Ocean University, Dalian 116023, China; 3 College of Marine and Biological Engineering, Yancheng Institute of Technology, Yancheng 224051, China; suiyanming@foxmail.com; 4 Co-Innovation Center of Jiangsu Marine Bio-Industry Technology, Jiangsu Ocean University, Lianyungang 222005, China; dzg7712@163.com; 5 Jiangsu Key Laboratory of Marine Genetic Resources and Breeding, Jiangsu Ocean University, Lianyungang 222005, China

**Keywords:** *Ruditapes philippinarum*, whole-genome resequencing, population genetic structure, selection signals

## Abstract

The Manila clam (*Ruditapes philippinarum*) is an economically important bivalve widely cultured in coastal aquaculture systems of China. However, the genome-wide genetic background and germplasm differentiation of geographically distinct populations remain unclear, which constrains germplasm conservation and the development of selective breeding programs. In this study, 50 individuals from five representative coastal populations (QZ, ZZ, ZP, CL, and NH) in China were subjected to whole-genome resequencing, producing 126.67 Gb of clean data and 92,593,087 SNPs after stringent filtering. Genetic diversity analyses showed that nucleotide diversity (π) ranged from 0.2453 to 0.2588, observed heterozygosity (*H*o) from 0.1316 to 0.1492, and expected heterozygosity (*H*e) from 0.2303 to 0.2435, with the CL population exhibiting relatively lower diversity. Population differentiation was low to moderate, with pairwise *F*_ST_ values ranging from 0.0454 to 0.0557. Principal component analysis, neighbor-joining phylogenetic analysis, and Admixture clustering consistently indicated limited population structure and extensive genetic admixture. TreeMix analysis further revealed directional gene flow among populations. Rapid linkage disequilibrium decay and predominantly positive Tajima’s D values suggested relatively stable demographic histories. Despite low genome-wide differentiation, combined *F*_ST_ and nucleotide diversity ratio analyses identified localized selective sweep signals in specific genomic regions. These results provide preliminary genome-wide insights into genetic diversity, population connectivity, and candidate localized differentiation signals in *R. philippinarum*. Given the low sequencing depth, the findings should be interpreted as population-level observations that require further validation using higher-depth genomic datasets before being applied to germplasm conservation and selective breeding programs.

## Simple Summary

The Manila clam (*Ruditapes philippinarum*) is an important shellfish widely cultured along the Chinese coast, contributing both to aquaculture production and coastal ecosystem health. Understanding the genetic diversity and population structure of this species is essential for conserving its genetic resources and improving breeding strategies. In this study, we analyzed the genomes of Manila clam populations from five coastal regions of China. The results showed that these populations maintain high genetic diversity and are strongly connected through gene flow, resulting in only minor differences among locations. Despite the overall similarity, several specific genomic regions showed localized differentiation signals, suggesting that certain populations have evolved traits suited to their local environments. These findings provide a clearer understanding of the genetic makeup and connectivity of Manila clam populations, offering valuable guidance for sustainable breeding, germplasm conservation, and the long-term development of the aquaculture industry.

## 1. Introduction

The Manila clam, *Ruditapes philippinarum*, is an economically important bivalve widely cultured in coastal China and broadly distributed along the northwestern Pacific coast [1], where it contributes substantially to aquaculture production and benthic ecosystem functioning [2]. Recently, the rapid expansion of aquaculture has markedly increased its production scale. However, large-scale seed translocation, unregulated stock mixing, and the lack of systematic germplasm management have raised concerns about the genetic integrity and increasing genetic homogenization among populations [3,4]. In addition, the coexistence of multiple cultured stocks and geographically distinct populations has further obscured the genetic background and germplasm relationships of *R. philippinarum*. Therefore, population genomic analyses of genetic diversity and population structure are essential for germplasm assessment and selective breeding strategies. However, the genetic background and population differentiation of geographically distinct *R. philippinarum* populations remain poorly understood. Genetic diversity and population structure are key determinants of population resilience to environmental change and long-term breeding potential. Genetic differentiation among populations reflects historical processes such as dispersal, gene flow, population divergence, and also provides foundations for germplasm conservation, resource management, and genetic improvement [5,6]. Previous genetic studies on *R. philippinarum* mainly relied on mitochondrial DNA and microsatellite markers to assess genetic diversity and population differentiation [7,8]. However, the limited marker density and genomic coverage of these traditional approaches constrained their ability to capture genome-wide genetic variation and to resolve fine-scale population structure or potential adaptive signals [9,10].

Population genomics approaches based on whole-genome resequencing and reduced-representation sequencing have been increasingly applied in non-model aquaculture species, offering higher resolution than traditional molecular markers [11,12]. In bivalve aquaculture species, including the Pacific oyster (*Crassostrea gigas*) [13], scallops and pearl oysters [14], genome-wide analyses identified geographic differentiation and adaptive selection signals. In other aquaculture species, such as grass carp (*Ctenopharyngodon idella*) and Pacific white shrimp (*Litopenaeus vannamei*), candidate genomic regions associated with growth performance and environmental adaptation were also detected [15,16]. Despite recent advances, genome-wide characterization of population structure and selection signals in *R. philippinarum* remains limited because previous studies primarily relied on low-density traditional markers with insufficient genomic coverage [17,18]. As a result, fine-scale genetic structure and genome-wide selection signals remained poorly resolved, restricting the effective use of genomic information for germplasm assessment and selective breeding.

In this study, we investigated five representative coastal populations of *R. philippinarum* along the Chinese coastline using whole-genome resequencing. Genome-wide SNPs were used to evaluate genetic diversity, population structure, and putative selective sweep regions associated with adaptive variation. Specifically, this study aimed to elucidate genetic diversity, assess population structure, and detect candidate genomic regions of *R. philippinarum*. The findings may provide useful genomic information for germplasm conservation and offer fundamental information for selective breeding and sustainable management of *R. philippinarum* aquaculture.

## 2. Materials and Methods

### 2.1. Sample Collection

**Figure 1:**
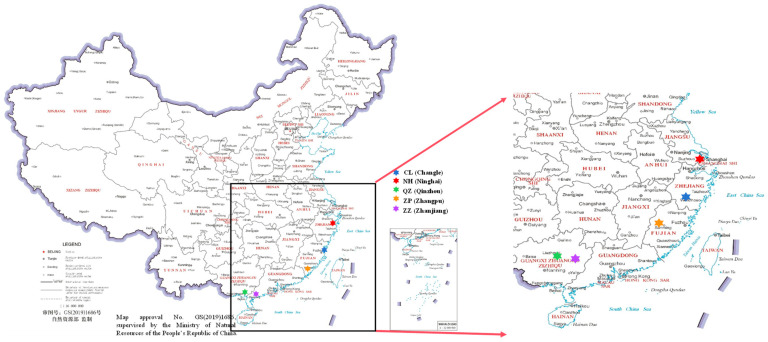
Sampling locations of the Manila clam (*R. philippinarum)*. Colored stars indicate the five sampled geographic populations along the Chinese coastline: Qinzhou (QZ), Zhanjiang (ZZ), Zhangpu (ZP), Changle (CL), and Ninghai (NH). The base map showing the topographic background of China and adjacent regions was obtained from the standard map service of the Ministry of Natural Resources of the People’s Republic of China (http://bzdt.ch.mnr.gov.cn/ (accessed on 10 May 2026), approval number GS(2019)1866).

50 individuals of *R. philippinarum* were collected from five representative coastal populations along the Chinese coastline in November 2024, with 10 individuals sampled from each population. All individuals were collected from wild populations in intertidal zones. The sampling locations included Qinzhou (QZ; 21°57′ N, 108°37′ E), Zhanjiang (ZZ; 21°16′ N, 110°21′ E), Zhangpu (ZP; 24°07′ N, 117°38′ E), Changle (CL; 25°57′ N, 119°31′ E), and Ninghai (NH; 29°17′ N, 121°26′ E) (Figure 1). The QZ, ZZ, and ZP populations were located along the southern and southeastern coasts of China, whereas the CL and NH populations were distributed along the East China Sea coast. After collection, mantle tissues were excised for genomic DNA extraction and immediately preserved at −80 °C.

### 2.2. DNA Extraction, Library Preparation, and Resequencing

Genomic DNA was extracted from mantle tissues of *R. philippinarum* using a standard phenol–chloroform protocol. DNA integrity and purity were evaluated by agarose gel electrophoresis and a NanoDrop 2000 spectrophotometer (Thermo Fisher Scientific, Waltham, MA, USA). High-quality genomic DNA was subsequently fragmented using a Covaris S220 ultrasonicator for library construction. Sequencing libraries were prepared using the TruSeq DNA PCR-Free Library Prep Kit (Illumina, San Diego, CA, USA) following the manufacturer’s instructions. Libraries with an average insert size of approximately 400 bp were generated and assessed using an Agilent 2100 Bioanalyzer (Agilent Technologies, Santa Clara, CA, USA) with the High Sensitivity DNA Kit. Library concentrations were quantified using the Quant-iT PicoGreen dsDNA Assay Kit (Thermo Fisher Scientific, USA), and those exceeding 2 nM were selected for sequencing. Qualified libraries with unique index sequences were pooled at equimolar ratios, denatured with NaOH, and subjected to paired-end sequencing (2 × 150 bp) on the Illumina NovaSeq 6000 platform.

### 2.3. Variant Calling, Annotation, and Quality Control

Raw sequencing data were subjected to quality control using AdapterRemoval v2.3.1 [19] to trim adapter sequences and remove low-quality reads. Reads containing more than 10% unidentified bases (N) or with over 50% of bases having a quality score < 5 were discarded, yielding high-quality clean reads. Clean reads were aligned to the *R. philippinarum* reference genome (NCBI assembly GCF_026571515.1) [20] with default parameters. Duplicate reads were removed, and alignment files were processed, including conversion, sorting, and indexing, using SAMtools v1.9 [21]. Variant calling was performed using GATK v3.8 [22]. Local realignment around insertion–deletion (InDel) regions was conducted using RealignerTargetCreator and IndelRealigner prior to variant detection. Single-nucleotide polymorphisms (SNPs) were identified using the UnifiedGenotyper with parameters *stand_call_conf* = 30 and *stand_emit_conf* = 10. To ensure SNP reliability, variants were filtered using the following criteria: Fisher strand bias (FS) ≤ 60, HaplotypeScore ≤ 13.0, Mapping Quality (MQ) ≥ 40, Quality by Depth (QD) ≥ 2.0, Read Position Rank Sum (ReadPosRankSum) ≥ −8.0, and Mapping Quality Rank Sum (MQRankSum) ≥ −12.5. Only SNPs passing all filters were retained for downstream analyses. To further improve SNP reliability, variants with minor allele frequency (MAF) < 0.05 were excluded from downstream analyses. SNP sites with missing genotype rates greater than 20% were also removed. No individuals were excluded based on missing genotype rates because all samples passed the predefined quality-control criteria. No additional genotype-level filtering based on genotype quality, genotype depth, or site depth was applied. Because the average sequencing depth per individual was relatively low, stringent SNP quality-control filtering and missing-data filtering were applied to reduce potential genotype uncertainty associated with low-coverage resequencing. All downstream population genomic analyses were conducted using the resulting filtered high-quality SNP dataset. The present study primarily focused on broad-scale population-level genomic patterns rather than high-confidence individual genotype inference. Functional annotation of SNPs was performed using ANNOVAR (version 2020-06-08) [23] based on the reference genome annotation. InDel variants were also detected using GATK v3.8 with the same calling parameters. InDel sites were extracted using SelectVariants and filtered using the following criteria: FS ≤ 200, read depth (DP) > 4, QD ≥ 2.0, and ReadPosRankSum ≥ −20. Sites with missing data were removed. The number and length distribution of InDel variants were subsequently summarized for downstream analyses.

### 2.4. Genetic Diversity and Population Structure Analyses

Based on the filtered high-quality SNP dataset, genetic diversity and population differentiation among five populations of *R. philippinarum* were evaluated. Genetic diversity indices, including nucleotide diversity (π), observed heterozygosity (*H*o), expected heterozygosity (*H*e), and inbreeding coefficient (*F*_IS_), were calculated to assess within-population genetic variation. Nucleotide diversity (π) was calculated based on the filtered high-quality SNP dataset and was primarily used for relative comparisons of genetic diversity among populations rather than absolute whole-genome per-site nucleotide diversity estimation. Population differentiation among populations was estimated using the fixation index (*F*_ST_). To infer phylogenetic relationships among individuals from different populations, a neighbor-joining (NJ) tree was constructed based on SNP data. Pairwise genetic distances were calculated using TreeBest v1.9.2, and node support was evaluated with 1000 bootstrap replicates. Principal component analysis (PCA) was conducted using GCTA v1.93.3. to assess genetic variation, and the first three principal components were used to visualize clustering patterns among individuals from different populations. Population genetic structure was further inferred using ADMIXTURE v1.3.0, with the number of genetic clusters (K) ranging from 2 to 10. The optimal K value was determined based on the minimum cross-validation (CV) error.

### 2.5. Linkage Disequilibrium, Tajima’s D, and Selective Sweep Analysis

Linkage disequilibrium (LD) patterns were evaluated for each population of *R. philippinarum* using high-quality SNP datasets. Pairwise LD (r^2^) between SNPs was calculated using PopLDdecay v3.41 [24], and LD decay curves were generated by plotting r^2^ against the physical distance between SNPs. Differences in LD decay among populations were subsequently compared. Tajima’s D statistics were calculated to assess deviations from neutral evolutionary expectations among populations. Genome-wide Tajima’s D values were estimated using a sliding-window approach implemented in VCFtools v0.1.16 [25], with a window size of 1,000,000 bp. The distribution of Tajima’s D values across the genome was examined for each population. Selective sweep signals were identified based on a combined analysis of population differentiation (*F*_ST_) and nucleotide diversity ratio (θπ ratio). Pairwise comparisons among populations were conducted independently using a sliding-window approach implemented in VCFtools, with a window size of 100 kb and a step size of 10 kb. Genome-wide *F*_ST_ values and θπ ratios were calculated independently for each pairwise comparison. In all pairwise comparisons, the first population listed was used as the numerator and the second population as the denominator. Genomic windows simultaneously exhibiting significantly high *F*_ST_ values (top 5%) and extreme θπ ratios (top 5%, either high or low after log_2_ transformation) were considered putative candidate regions under selection. Candidate windows identified from different pairwise comparisons were treated independently and were not merged across comparisons. Genes located within these regions were identified as candidate genes potentially associated with selection.

## 3. Results

### 3.1. Whole-Genome Resequencing, Quality Control and Variant Discovery

**Figure 2:**
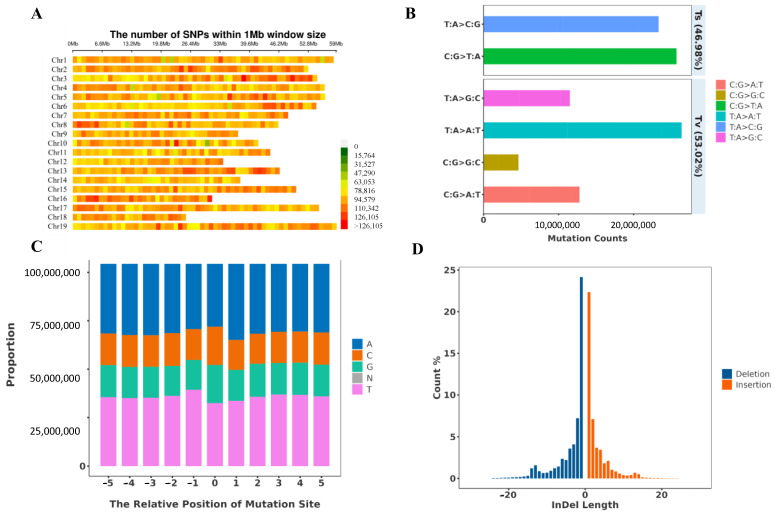
Genome-wide characteristics of SNP and InDel variants in the Manila clam (*R. philippinarum*). (**A**) Distribution of SNsP density across 19 chromosomes based on 1-Mb sliding windows. (**B**) Frequencies of SNP mutation types, including transitions (Ts) and transversions (Tv). (**C**) Base composition in the flanking regions surrounding SNP sites (−5 to +5 bp). (**D**) Length distribution of insertion and deletion (InDel) variants.

Whole-genome resequencing of 50 *R. philippinarum* individuals generated 852,111,000 high-quality reads, yielding 126.67 Gb of clean sequencing data with an average sequencing depth of 1.75× per individual (Table S1). Detailed sequencing quality-control statistics for each individual, including mapping rate and sequencing quality metrics, are summarized in Table S1. After quality filtering and variant calling, 92,593,087 high-quality SNPs were identified for subsequent analyses. Based on a 1-Mb sliding-window analysis, SNPs were distributed across all 19 chromosomes of *R. philippinarum* and illustrated a continuous but uneven genomic distribution (Figure 2A). Although SNP density varied among chromosomes and genomic regions, no apparent SNP deserts or highly enriched regions were detected. In addition, Transition and transversion mutations accounted for 46.98% and 53.02%, respectively, resulting in a relatively low Ts/Tv ratio (Figure 2B). Flanking base composition around SNP sites remained relatively stable within ±5 bp of mutation sites (Figure 2C). InDel analysis indicated that both insertion and deletion variants were predominantly 1 bp in length, with variant frequency decreasing as InDel length increased (Figure 2D).

### 3.2. Population Genetic Diversity Analysis

Based on the genome-wide SNP dataset, genetic diversity indices indicated detectable genetic variation among *R. philippinarum* populations (Table 1). SNP-based nucleotide diversity estimates (π), calculated from the filtered high-quality SNP dataset, ranged from 0.2453 to 0.2588 and were used primarily for relative comparisons among populations rather than as absolute whole-genome per-site nucleotide diversity estimates. The lowest value was observed in the CL population and the highest in the ZZ population. Observed heterozygosity (*H*o) ranged from 0.1316 to 0.1492, while expected heterozygosity (*H*e) ranged from 0.2303 to 0.2435 and was consistently higher than *H*o in all populations. Correspondingly, positive inbreeding coefficients (*F*_IS_) were detected in all populations, ranging from 0.3070 to 0.3204. The highest *F*_IS_ value occurred in the CL population (0.3204), whereas the ZP population exhibited the lowest value (0.3070), suggesting an apparent heterozygote deficiency. However, the elevated *F*_IS_ values may partially reflect heterozygote undercalling associated with low-coverage resequencing and genotype uncertainty.

### 3.3. Population Structure and Genetic Differentiation

**Figure 3:**
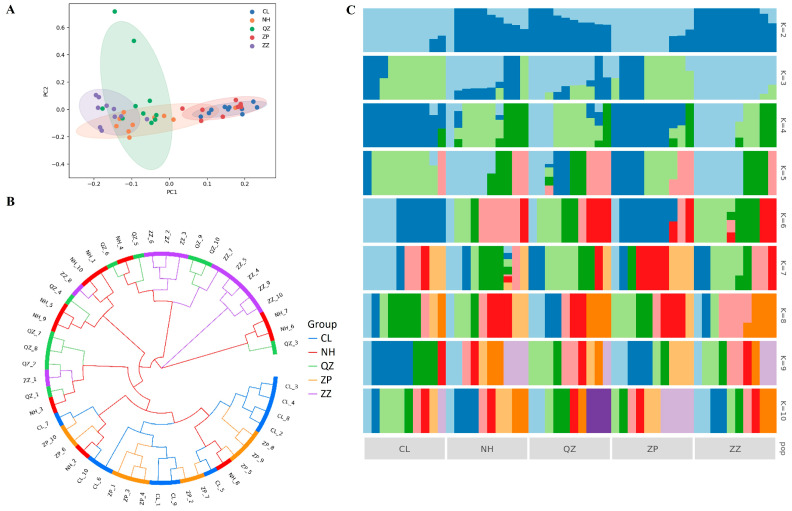
Population genetic structure of the Manila clam (*R. philippinarum*) inferred from genome-wide SNP data. (**A**) Principal component analysis (PCA) based on genome-wide SNPs. PC1 and PC2 explain 7.16% and 5.60% of the total genetic variation, respectively. (**B**) Neighbor-joining (NJ) phylogenetic tree showing genetic relationships among individuals from different populations. (**C**) Population structure inferred using Admixture (K = 2–10), showing the ancestral composition of each individual. Each column represents an individual, and the proportion of each color indicates the estimated contribution of a specific ancestral genetic component to that individual’s genome. Population labels are shown at the bottom of the figure.

Principal component analysis (PCA), neighbor-joining (NJ) phylogenetic analysis, and Admixture consistently indicated weak population structure among *R. philippinarum* populations. PCA revealed substantial overlap among individuals, with no clear population-specific clustering along the first two principal components (PC1 and PC2) (Figure 3A). Additional principal components did not reveal obvious further population differentiation beyond the weak structure observed in PC1 and PC2. Similarly, individuals from different populations were interspersed across branches in the NJ tree rather than from distinct geographic clades (Figure 3B). Admixture analysis also revealed extensive mixed ancestry across populations over the range of K values examined (Figure 3C). Pairwise genetic differentiation indices (*F*_ST_) further quantified genetic divergence among populations. *F*_ST_ values ranged from 0.0454 to 0.0557 (Table 2), indicating low genetic differentiation. The highest *F*_ST_ value was observed between the ZZ and CL populations (0.0557), whereas the lowest occurred between the ZP and CL populations (0.0454).

### 3.4. Potential Gene Flow Among Populations

**Figure 4:**
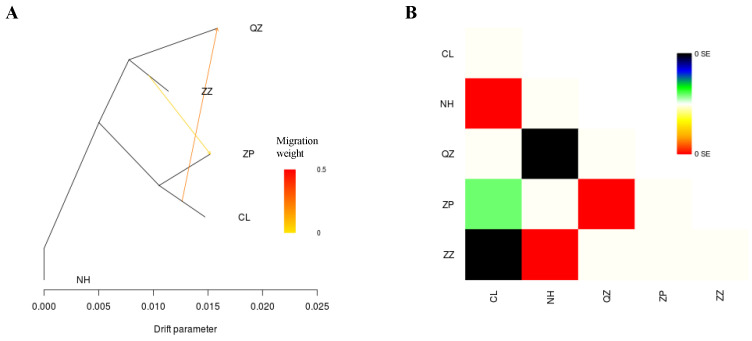
Gene flow among *R. philippinarum* populations inferred using TreeMix. (**A**) Maximum-likelihood phylogenetic tree based on genome-wide SNP data. Colored arrows indicate inferred migration events, with arrow direction representing gene flow and color intensity reflecting migration weight. The horizontal axis represents genetic drift. (**B**) Residual heatmap of the TreeMix model showing differences between observed and predicted allele frequency covariance.

TreeMix analysis suggested potential gene flow among *R. philippinarum* populations based on the genome-wide SNP dataset. The maximum-likelihood TreeMix tree was broadly consistent with the population structure inferred from PCA and NJ analyses (Figure 4A). Several putative migration edges were inferred among populations, including from CL to ZP and from QZ to ZZ, indicating possible genetic exchange among geographically separated populations. An additional migration signal was inferred between ZP and ZZ, suggesting further genetic connectivity. Residual fit analysis showed that the TreeMix model generally captured the covariance structure of allele frequencies among populations, although slightly elevated residuals were observed for a few population pairs, including ZZ–CL and QZ–NH (Figure 4B).

### 3.5. Linkage Disequilibrium (LD) Decay Analysis

**Figure 5:**
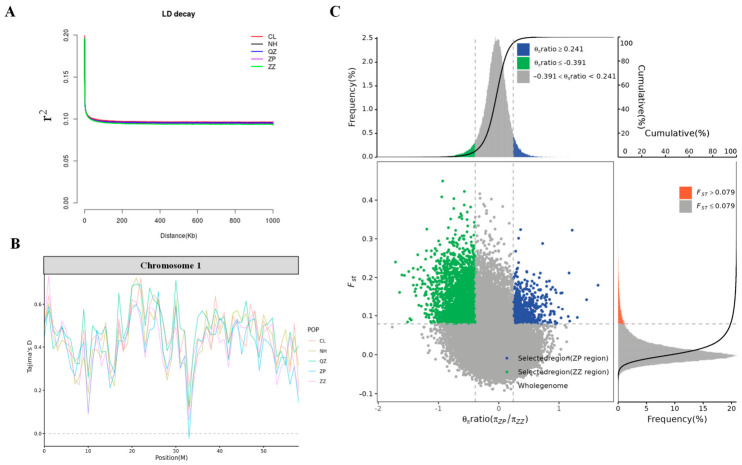
Linkage disequilibrium decay, neutrality test, and selective sweep signals in geographically distinct populations of the Manila clam (*R. philippinarum*). (**A**) Linkage disequilibrium (LD) decay patterns of five populations (CL, NH, QZ, ZP, and ZZ) based on genome-wide SNP data, showing the relationship between LD coefficient (r^2^) and physical distance. (**B**) Distribution of Tajima’s D values along Chromosome 1 calculated using a 1-Mb sliding window for the five populations. (**C**) Representative putative selective sweep analysis for the ZP–ZZ comparison based on combined *F*_ST_ and θπ ratio statistics. The θπ ratio was calculated as π(ZP)/π(ZZ), and log2 transformation was applied prior to threshold determination and candidate region identification.

LD decay patterns were compared among *R. philippinarum* based on the filtered high-quality SNP dataset. All *R. philippinarum* populations showed relatively high r^2^ values at short physical distances, followed by a rapid decline with increasing SNP distance (Figure 5A). At larger distances, r^2^ values gradually stabilized, indicating a typical LD decay pattern. The LD decay curves showed similar overall shapes among populations, suggesting comparable genome-wide LD patterns. Slight differences were observed in initial r^2^ levels and decay rates at short physical distances, where the LD curves of the CL, NH, QZ, ZP, and ZZ populations showed moderate separation. However, the curves gradually converged as physical distance increased, ultimately reaching similar stabilized r^2^ levels. These results indicated rapid LD decay across all *R. philippinarum* populations.

### 3.6. Distribution Patterns of Tajima’s D

Genome-wide Tajima’s D values showed consistent distribution patterns across the five populations (CL, NH, QZ, ZP, and ZZ) (Figure 5B). Most genomic regions exhibited positive Tajima’s D values, primarily ranging from 0.3 to 0.6, with similar patterns observed among populations. On Chromosome 1, Tajima’s D curves showed comparable fluctuation patterns along the chromosome. No extended genomic regions with persistently negative Tajima’s D values were detected. Only a few localized windows exhibited Tajima’s D values close to zero or slightly negative, and these signals were not consistently observed across populations. Genome-wide Tajima’s D values were consistently positive across most genomic regions in all populations.

### 3.7. Integrated Analysis of Selection Signals Based on F_ST_ and θπ Ratio

Selective sweep analyses were conducted for all pairwise population comparisons. Figure 5C shows the ZP–ZZ comparison as a representative example of the genome-wide distribution patterns of *F*_ST_ and θπ ratio values. Most genomic windows were concentrated within intermediate ranges, with only a small proportion located at the distribution tails. Windows with θπ ratios ≥0.241 or ≤−0.391 and FST values >0.079 (top 5%) were defined as putative candidate regions showing differentiation between populations. The threshold values of 0.241 and −0.391 corresponded to the upper and lower 5% tails of the empirical log2-transformed θπ ratio distribution, respectively. Most genomic windows were characterized by low *F*_ST_ values and moderate θπ ratios, indicating limited genome-wide differentiation. In contrast, a subset of windows exhibiting both elevated *F*_ST_ values and extreme θπ ratios formed distinct outlier clusters and were identified as putative selective sweep regions.

## 4. Discussion

### 4.1. Genetic Diversity, Population Connectivity, and Implications for Germplasm Management

Genome-wide SNP analyses suggest that geographically distinct populations of *R. philippinarum* maintain detectable SNP-based genetic diversity while exhibiting low overall genetic differentiation. Because nucleotide diversity (π) was estimated from filtered SNP loci, these values should be interpreted primarily as relative indicators of genetic diversity among populations. Similar patterns have also been observed in marine bivalves and East Asian populations of *R. philippinarum* based on genome-wide analyses [1]. This pattern may be associated with large effective population sizes and ongoing gene flow and may provide a basis for germplasm utilization and breeding improvement. Consistently, PCA, phylogenetic reconstruction, Admixture, and TreeMix analyses indicated weak population structure and relatively high connectivity, with low pairwise *F*_ST_ values suggesting limited differentiation among populations [25]. Such connectivity may be influenced by both natural dispersal and aquaculture-related activities, including cross-regional seed translocation and stock enhancement. Comparable patterns of weak population differentiation have also been reported in geographically distinct *R. philippinarum* populations using microsatellite markers [7]. Despite limited genome-wide differentiation, divergence may still occur in specific genomic regions, where selection may act more strongly under high connectivity [26,27,28,29]. These localized signals may represent potential targets for genetic improvement. From a germplasm management perspective, genetic connectivity may help maintain diversity and reduce inbreeding risk, but excessive gene flow may weaken population-specific characteristics and constrain the development of distinct breeding lines [30]. Therefore, breeding strategies for *R. philippinarum* should balance genetic exchange with the conservation of population-specific variation.

### 4.2. Genetic Signals of Population History and Potential Population Stability

Tajima’s D analyses showed predominantly positive values across the genome in all populations, with generally consistent patterns among groups. Under neutral theory, such patterns may reflect an excess of intermediate-frequency alleles and can be associated with multiple processes, including balancing selection, population structure, demographic history, or technical effects associated with low-coverage resequencing [31]. No clear signals of recent expansion or severe bottlenecks were detected, as strongly negative Tajima’s D values and elevated linkage disequilibrium were not observed [32]. Consistently, all populations exhibited relatively rapid LD decay and low long-range LD, which may be associated with large effective population sizes and frequent recombination [33]. Together, these patterns may be consistent with relatively stable demographic signals, but they should not be interpreted as definitive evidence of population stability. Additional demographic analyses are required to distinguish among alternative explanations [34].

### 4.3. Localized Selection Signals, Genetic Basis of Environmental Adaptation, and Practical Implications

Although overall genetic differentiation among *R. philippinarum* populations was low, combined *F*_ST_ and θπ ratio analyses identified selective sweep signals in several localized genomic regions. This pattern suggests that divergence may still occur in specific regions despite high gene flow and limited genome-wide differentiation [35]. In species with high connectivity, gene flow may constrain genome-wide divergence, whereas selection may act on a limited number of genomic regions. Environmental heterogeneity in coastal ecosystems may further contribute to localized genomic divergence in marine organisms, potentially leading to localized differentiation. The selective signals identified here are therefore consistent with the generally low *F*_ST_ values observed across the genome. Although such signals may potentially reflect selective processes, alternative explanations such as background selection or demographic effects cannot be excluded [36]. From a germplasm perspective, these localized signals may represent more informative targets than genome-wide differentiation for genetic improvement in highly connected species [30]. One limitation of the present study is the relatively low sequencing depth per individual (average 1.75×). Although low-coverage resequencing may still recover broad-scale population structure and allele frequency patterns when sufficient genome-wide SNPs and sample sizes are available, low sequencing depth may increase genotype uncertainty and potentially influence heterozygosity estimation, *F*_IS_ values, Tajima’s D statistics, and selective sweep detection. Therefore, the selective sweep signals identified in this study should be interpreted as putative candidate regions requiring further validation using higher-depth resequencing or targeted genotyping approaches. The relatively low Ts/Tv ratio observed in this study may partially reflect increased genotype uncertainty associated with low-coverage resequencing. Therefore, all population-genomic inferences presented in this study should be regarded as preliminary population-level observations rather than definitive estimates of population genetic parameters. While the genome-wide SNP dataset provides useful information regarding broad-scale patterns of genetic diversity and connectivity, higher-depth resequencing, genotype likelihood-based analyses, and independent validation datasets will be necessary to confirm the robustness of the observed patterns and candidate selective regions.

## 5. Conclusions

Based on low-coverage whole-genome resequencing data, the five *R. philippinarum* populations examined in this study showed broadly similar genome-wide patterns, including relatively high genetic diversity, weak population structure, and evidence of substantial genetic connectivity. Population structure analyses suggested limited geographic differentiation and extensive admixture among populations. Tajima’s D and linkage disequilibrium decay analyses revealed patterns that may be consistent with relatively stable demographic histories, although these observations should be interpreted cautiously given the low sequencing depth and potential genotype uncertainty. In addition, several candidate genomic regions showing localized differentiation signals were identified despite the overall low level of genome-wide differentiation. However, these candidate regions and their potential biological significance require further validation using higher-depth resequencing or targeted genotyping approaches. Overall, this study provides preliminary genome-wide evidence for high population connectivity and candidate localized differentiation signals in *R. philippinarum* and offers a useful foundation for future population genomic research, germplasm conservation, and breeding resource evaluation.

## Figures and Tables

**Table 1:** Summary of genetic diversity indices for five *R. philippinarum* populations. SNP-based π values were calculated from filtered high-quality SNP loci and used for relative comparisons among populations.

Pop ID	Num Indv	*H*o	*H*e	Filtered SNP-Based π	*F*_IS_
QZ	10	0.1444	0.2418	0.2574	0.3161
ZP	10	0.1413	0.2343	0.2492	0.3070
ZZ	10	0.1492	0.2435	0.2588	0.3088
CL	10	0.1316	0.2303	0.2453	0.3204
NH	10	0.1455	0.2427	0.2581	0.3169

**Table 2:** Pairwise population differentiation (*F*_ST_) among five *R. philippinarum* populations.

pop	QZ	ZP	ZZ	CL	NH
QZ	—	0.0514	0.0458	0.0531	0.0465
ZP	—	—	0.0534	0.0454	0.0491
ZZ	—	—	—	0.0557	0.0455
CL	—	—	—	—	0.0509

## Data Availability

The raw whole-genome resequencing data generated in this study have been deposited in the NCBI Sequence Read Archive (SRA) under BioProject accession PRJNA1474381. The filtered SNP dataset, associated metadata, and supplementary materials supporting the findings of this study are publicly available in Figshare at https://doi.org/10.6084/m9.figshare.32567553. Analysis scripts are available from the corresponding author upon reasonable request.

## Supplementary Materials

The following supporting information can be downloaded at: https://www.mdpi.com/article/10.3390/ani16121897/s1, Table S1: Sample sequencing results and genome mapping rate statistics.
