# Supplementary material for: Genome-Wide Resequencing Reveals High Connectivity and Localized Adaptive Signals in Manila Clam (Ruditapes philippinarum) Populations Along the Southeastern Coast of China
Source: Animals (Basel). 2026 Jun 18;16(12):1897. doi: 10.3390/ani16121897 (PMC13295439; doi:10.3390/ani16121897)
Supplement: Supplementary file 1 [file animals-16-01897-s001.zip › animals-4340609-supplementary.pdf]

Table S1. Sample sequencing results and genome mapping rate statistics

Sample Name	HQ_Reads	HQ_Data(bp)	GC(%)	Q20(%)	Q30(%)	Gropu	Mapping
CL_1	13,908,138	2,060,411,049	34.42	97.28	95.14	CL	96.24%
CL_2	16,413,154	2,437,475,606	33.88	97.42	95.39	CL	96.34%
CL_3	14,474,692	2,149,146,383	33.89	97.3	95.17	CL	96.03%
CL_4	15,806,266	2,351,999,390	33.96	97.44	95.42	CL	96.18%
CL_5	18,398,892	2,736,348,813	33.75	97.41	95.36	CL	95.96%
CL_6	14,626,062	2,170,322,660	34.09	97.25	95.08	CL	96.16%
CL_7	17,235,474	2,564,140,266	33.54	97.45	95.44	CL	96.03%
CL_8	16,882,044	2,500,356,428	34.34	97.29	95.19	CL	96.47%
CL_9	16,666,894	2,478,320,488	33.79	97.4	95.34	CL	96.06%
CL_10	16,046,790	2,388,042,561	33.55	97.54	95.6	CL	95.79%
NH_1	15,454,508	2,294,388,398	34.09	97.4	95.36	NH	96.17%
NH_2	18,424,366	2,730,873,531	34.2	97.31	95.2	NH	96.19%
NH_3	15,441,376	2,296,205,689	33.77	97.53	95.6	NH	96.42%
NH_4	17,969,292	2,667,152,013	34	97.43	95.4	NH	96.26%
NH_5	20,533,320	3,054,316,537	33.68	97.44	95.43	NH	96.10%
NH_6	19,173,406	2,856,015,331	33.28	97.64	95.8	NH	96.11%
NH_7	18,519,842	2,743,837,616	33.97	97.35	95.27	NH	96.18%
NH_8	16,418,030	2,440,493,599	33.79	97.48	95.51	NH	96.31%
NH_9	17,756,412	2,642,169,860	33.62	97.51	95.53	NH	96.11%
NH_10	15,628,804	2,328,091,251	33.57	97.59	95.67	NH	96.24%

QZ_1	14,929,100	2,220,709,288	33.71	97.36	95.26	QZ	96.04%
QZ_2	15,023,870	2,233,341,672	33.64	97.38	95.3	QZ	95.62%
QZ_3	15,031,492	2,233,266,800	33.81	97.38	95.32	QZ	96.02%
QZ_4	14,569,440	2,167,185,255	33.33	97.36	95.27	QZ	95.68%
QZ_5	16,026,508	2,374,440,390	34.08	97.3	95.17	QZ	96.09%
QZ_6	18,272,946	2,719,049,318	33.24	97.55	95.6	QZ	95.46%
QZ_7	22,859,166	3,396,379,218	33.71	97.48	95.5	QZ	96.18%
QZ_8	14,491,710	2,151,132,940	34.04	97.24	95.05	QZ	94.55%
QZ_9	17,496,318	2,600,251,874	33.69	97.33	95.22	QZ	95.84%
QZ_10	16,047,250	2,386,298,853	33.5	97.23	95.03	QZ	95.60%
ZP_1	20,550,398	3,056,119,214	33.73	97.54	95.61	ZP	96.18%
ZP_2	17,792,174	2,645,983,495	33.48	97.5	95.53	ZP	96.16%
ZP_3	19,572,520	2,911,773,379	33.51	97.58	95.68	ZP	96.14%
ZP_4	17,080,434	2,542,417,002	33.47	97.57	95.66	ZP	96.04%
ZP_5	17,678,002	2,626,708,647	33.7	97.4	95.35	ZP	96.03%
ZP_6	15,972,104	2,379,653,944	33.29	97.6	95.69	ZP	96.08%
ZP_7	16,820,338	2,502,472,570	33.65	97.45	95.43	ZP	96.04%
ZP_8	15,814,444	2,355,624,918	33.36	97.45	95.43	ZP	95.85%
ZP_9	16,529,886	2,459,208,944	33.35	97.42	95.38	ZP	95.61%
ZP_10	13,421,432	1,994,839,240	33.52	97.09	94.75	ZP	95.29%
ZZ_1	17,838,824	2,655,243,826	33.48	97.51	95.54	ZZ	95.71%
ZZ_2	15,790,050	2,349,434,455	33.6	97.38	95.32	ZZ	94.67%

ZZ_3	18,312,266	2,721,522,737	33.59	97.43	95.41	ZZ	95.20%
ZZ_4	22,678,722	3,372,953,344	33.65	97.55	95.62	ZZ	95.89%
ZZ_5	19,315,436	2,874,145,897	33.54	97.57	95.64	ZZ	96.05%
ZZ_6	14,843,300	2,205,428,484	33.61	96.93	94.47	ZZ	95.06%
ZZ_7	16,010,796	2,377,050,711	33.87	97.11	94.82	ZZ	95.15%
ZZ_8	16,799,994	2,498,339,841	33.9	97.46	95.47	ZZ	92.25%
ZZ_9	20,215,104	3,010,856,673	33.54	97.61	95.72	ZZ	95.81%
ZZ_10	18,549,214	2,757,641,769	33.6	97.36	95.28	ZZ	95.65%
**Total**	852,111,000					**Average:**	0.958258
